# Effect of Incorporated ZnO Nanoparticles on the Corrosion Performance of SiO_2_ Nanoparticle-Based Mechanically Robust Epoxy Coatings

**DOI:** 10.3390/ma13173767

**Published:** 2020-08-26

**Authors:** Ubair Abdus Samad, Mohammad Asif Alam, Arfat Anis, El-Sayed M. Sherif, Sulaiman I. Al-Mayman, Saeed M. Al-Zahrani

**Affiliations:** 1 Center of Excellence for Research in Engineering Materials (CEREM), King Saud University, P.O. Box 800, Riyadh 11421, Saudi Arabia; uabdussamad@ksu.edu.sa (U.A.S.); esherif@ksu.edu.sa (E.-S.M.S.); 2 SABIC Polymer Research Center (SPRC), Chemical Engineering Department, King Saud University, P.O. Box 800, Riyadh 11421, Saudi Arabia; szahrani@ksu.edu.sa; 3 Electrochemistry and Corrosion Laboratory, Department of Physical Chemistry, National Research Centre, El-Behoth St. 33, Dokki, Cairo 12622, Egypt; 4 Water and Energy Research Institute (WERI), King Abdulaziz City for Science and Technology (KACST), P.O. Box 6086, Riyadh 11442, Saudi Arabia; smayman@kacst.edu.sa

**Keywords:** epoxy, coatings, EIS, silica, ZnO, nanoparticles, corrosion

## Abstract

This paper presents the studies of the development of a high-performance epoxy coating for steel substrates. To this end, it investigated the synergistic effect of incorporating zinc oxide (ZnO) nanoparticles into nanosilica containing epoxy formulations. The mechanical properties of the epoxy coating formulations were improved by modifying the surfaces of the silica nanoparticles (5 wt.%) with 3-glycidoxypropyl trimethoxysilane, which ensured their dispersal through the material. Next, the ZnO nanoparticles (1, 2, or 3 wt.%) were incorporated to improve the corrosion performance of the formulations. The anticorrosive properties of the coatings were examined by electrochemical impedance spectroscopy (EIS) of coated mild steel specimens immersed in 3.5% NaCl solution over different time intervals (1 h to 30 days). Incorporation of the ZnO nanoparticles and the nanosilica into the coating formulation improved the corrosion resistance of the epoxy coating even after long-term exposure to saline test solutions. Finally, to evaluate how the nanoparticles affected the chemical and morphological properties of the prepared coatings, the coatings were characterized by scanning electron microscopy (SEM), Fourier transform infrared (FTIR) spectroscopy, and X-ray diffraction (XRD).

## 1. Introduction

Historically, different types of coatings have been used according to the area of application for which they are developed, and there has been continuous advancements in these areas. Traditionally, metal, ceramic, and polymeric coatings are used in biomedical applications, cutting tools, batteries, heavy industrial equipment, pipes and fittings for offshore applications, etc., to enhance the life of base materials or to increase the instruments’ work efficiency. Ibrahim et al. [1] developed a Mg-based ceramic coating and coated it onto medical implants to provide strength and corrosion resistance. Coating was done using plasma electrolytic oxidation (PEO) and a sol–gel (layer-by-layer) techniques. The results indicate an increased corrosion resistance and loss in mechanical strength of only 3% compared to 30% loss for uncoated alloys under similar conditions. Ramezanzadeh et al. [2] developed a new type of protective coating with anticorrosion properties using a one-pot synthesis method of zeolitic imidazolate framework-8 (ZIF-8) on graphene oxide (GO) sheets mixed with epoxy resin. Prepared steel samples were exposed to a NaCl solution, and the results indicated an increase in inhibiting efficiency of 79% for nanoparticle-modified GO with the ZIF-8 process compared to neat GO.

Offshore metallic structures are coated with multilayer materials to prevent corrosion and to achieve the required properties. A coating layer generally consists of a primer, an intermediate, and a top coat [3]. The primer improves the adhesion and protects the steel substrate from corrosion. The intermediate layer provides the adhesion between the top coat and primer and blocks aggressive species from the surface. Finally, the top coat confers the mechanical properties such as resistance to scratching, impact, and abrasion, and the environmental properties such as color, gloss, and protection against ultraviolet (UV) radiation [4].

During the last few decades, coating industries have relied heavily on toxic heavy metal pigments such as chromates [5,6], which are gradually being replaced by more environmentally friendly pigments. Inhibitors based on heavy metal compounds were widely used in classical anticorrosive paints, but their use is now restricted to reduce environmental contamination and health risks to humans [7]. Environmental regulations in different countries are promoting active research on lowly toxic alternative pigments with the same performance as classical pigments.

Anticorrosion epoxy coatings act as a barrier and an inhibitor. The barrier effect excludes the transport of aggressive species to the substrate surface, whereas the inhibiting effect is conferred by inhibiting pigments or chemical conversion of the layer by passivation, which require a huge quantity of hazardous compounds. Epoxy coatings often form a high cross-linking density that increases the anticorrosion performance. These coatings function by reducing the mobility of the polymer chains; hence, the free volume through which destructive species can penetrate the coatings [8].

Most researchers working on coatings are attempting to reduce the use of toxic heavy metal pigments in multilayer systems. The development of less hazardous coating systems requires a significantly different approach to traditional development. In recent years, researchers have attempted to reduce the volatile organic contents in paint formulations and to improve the corrosion resistance of the coating by incorporating nanoparticles. This paper explores the synergistic effect of different nanoparticles to enhance the anticorrosion behavior and the mechanical properties of an epoxy coating system for steel substrates.

The incorporation of nano-ZnO and nanosilica obtains self-cleaning epoxy coatings with desirable properties, such as easy sliding of water droplets. Nanosilica pigment confers nano-roughness surfaces and antifouling characteristics [9]. Other researchers have incorporated nanosilica, ZnO, alumina, and similar additives to improve the mechanical strength of epoxy coatings, provide anticorrosion properties, and block UV light [10]. SiO_2_ nanoparticles are extensively used as scratch-resistant agents in the paint industry. They also provide water repellency properties that not only protect against corrosion but also produce a glossy finished surface [11]. With their large surface area, nanoparticles are popular additives in anticorrosion protection, because they function as molecular-corrosion inhibitor carriers [12].

Shi et al. [13] analyzed nanoparticles of SiO_2_, zinc, iron oxide (Fe_2_O_3_), and halloysite clay (Al_2_Si_2_O_5_(OH)_4_·2H_2_O + SiO_2_) (surface area = 2 cm^2^). They incorporated 1 wt.% of the nanoparticles into bisphenol A diglycidyl ether (DGEBA) and cured the mixture with aliphatic polyamine at a weight ratio of 2:1. The electrochemical properties of the coating layer were improved even after exposure to 3.5% NaCl solution for 28 days. In a saline solution (3 wt.%) for 28 days, SiO_2_ particles incorporated at 1 wt.% retarded the corrosion rate of an epoxy coating on steel by 32 times, relative to the unmodified base coating. The nano-SiO_2_ occupies the pores in the epoxy network and bridges the molecules in the interconnected matrix, enhancing the cross-linking density of the cured epoxy, thereby improving its corrosion protection on steel substrates.

Behzadnasab et al. [14] reported that 3 wt.% nano-zirconium dioxide (with an average particle size of 15 nm) delivers promising anticorrosion behavior, with a coating resistance of 20 × 10^9^ Ω cm^2^. They incorporated nano-zirconium dioxide modified with amino propyl trimethoxy silane into an epoxy matrix (DGEBA), followed by curing with amines at a weight ratio of 2:1. The weight percentage of the nanoparticles was varied as 1, 2, and 3 wt.%. After five days of immersion, the Nyquist plot of the neat epoxy showed the typical semicircle at high frequencies and a second semicircle at lower frequencies, indicating the start of corrosion by water penetration and ionic charge movement through the coating layer. After 30–60 days of immersion, the Nyquist plot of the epoxy coating with 1% nano zirconium dioxide also developed a second semicircle, but its resistance remained above 1.2 × 10^9^ Ω cm^2^. Meanwhile, the Nyquist plots of the coatings with 2 and 3 wt.% zirconium dioxide showed a single capacitive loop after 120 days of immersion, although the resistance tended to decrease over time. The stability and high resistance over a prolonged period of exposure confirmed the efficiency and barrier properties of the ZrO_2_ particles.

Ramezanzadeh and Attar [15] incorporated zinc oxide (ZnO) nanoparticles at different weight percentages into a DGEBA epoxy matrix, followed by curing with a polyamide hardener. They found that when added at 3.5 or 5.0 wt.%, the ZnO nanoparticles significantly improved the coating’s corrosion resistance. The high surface area of the nano-sized ZnO particles increased the barrier properties of the film. However, when the proportion increased to 6.5 wt.%, further improvement was prevented by agglomeration of the nanoparticles [16].

The above studies provide cumulative evidence that nano-sized particles improve the anticorrosive properties of epoxy coatings. We recently reported that nanosilica-based epoxy coatings confer good anticorrosive resistance to stainless steel substrates, but their performance gradually deteriorated when immersed in saline test solutions for long periods [17]. Among the investigated coatings, epoxy with 5% nanosilica content achieved the best thermal, abrasion, and mechanical properties [18]. There is an increasing demand for coatings providing long-term corrosion protection of steel substrates. In this study, we investigated whether nano-ZnO confers any synergistic effect that improves the long-term anticorrosion behavior of epoxy coatings reinforced with 5 wt.% nanosilica.

The cross-linked structure and composition of these coatings were studied by X-ray diffraction (XRD) analysis and Fourier transfer infrared (FTIR) spectroscopy. The effect of incorporating nano-ZnO into the epoxy coating, and the anticorrosion properties of the prepared coatings, were elucidated by electrochemical impedance spectroscopy (EIS). The morphological features and distribution of the nanoparticles were revealed by scanning electron microscopy (SEM) and energy-dispersive X-ray spectroscopy (EDX).

## 2. Materials and Methods

The main formulating ingredient was Epikote 1001 resin procured from Hexion Chemicals (Iserlohn, Germany). Resin cross-linker (D-450 BD) was procured from Huntsman Advance Materials (Deutschland, Germany). The SiO_2_ and ZnO nanoparticles were acquired from Sigma-Aldrich (Catalog numbers 637,238 and 677,450, respectively, St. Louis, MO, USA). The solvents were methyl isobutyl ketone (MIBK), acetone, and xylene, all purchased from a Saudi local market.

Coating formulations were prepared with variable weight percentages (wt.%) of ZnO and a fixed wt.% of silica nanoparticles. The composition in wt.% of each formulation is given in Table 1. All formulations contained bisphenol A-based epoxy resin as the main constituent, along with other compatible ingredients such as solvents, air release agent, and epoxy resin cross-linker (D-450). Aided by xylene, the viscosity of the epoxy resin was reduced in a mechanical mixer (Sheen S2 disperse master, Sheen Instruments, Surrey, UK) operated at 500 rpm for five minutes. After this time, the other ingredients (except the nanoparticles) were added sequentially at the same operating speed. Initially, the air release agent was added to improve the mixing with the other formulating ingredients. Meanwhile, the nanoparticles were disseminated in acetone using the sonication technique in the presence of silane. The nanoparticle mixture was sonicated for 40 min to maximally disperse the nanoparticles. Once the sonication process had finished, the nanoparticle solution was poured dropwise into the diluted epoxy resin. Finally, the mixture was stirred thoroughly at 5000 rpm for 45 min to obtain a homogeneous dispersion, followed by a leftover time of 10 min for stabilization. After stabilization, the hardener was added to avoid air traps. To evaluate the electrochemical properties of the prepared coatings’ formulations, the formulations were coated onto the steel substrates of different sizes. In all the formulations, the silica nanoparticle content was fixed to 5 wt.%; at higher silica nanoparticles (6 wt.%), the problem of dispersion was encountered, leading to the formation of aggregates. This resulted in the deterioration of the coating properties. As shown in our previous study [17], the best results were obtained for 5 wt.% silica nanoparticle addition; therefore, in this study, we took this silica content as a reference. We further added the ZnO nanoparticles to these formulations to study if the addition of these nanoparticles showed any synergistic effect on the properties of the resulting coatings.

The presence of nanoparticles in the prepared coatings was verified by XRD using a Bruker (D8 Discover, Karlsruhe, Germany) diffractometer with Cu Kα radiation operated at 40 kV and 40 mA. The scanning speed was 2°/min and the range was 2*θ* = 10–80° at room temperature. The reaction between the epoxy and cross-linker in the presence of nanoparticles was investigated by FTIR.

The anticorrosion behavior of the epoxy coatings was determined by EIS. The three-electrode cell contained a Ag/AgCl reference electrode, a stainless steel sheet as the counter electrode, and steel panels coated with epoxy as the working electrode. The coatings were immersed in a 3.5% NaCl solution for different exposure periods (1 h to 30 days), and the EIS was performed by an Autolab Ecochemie PGSTAT 30 (Metrohm Autolab B.V., Amsterdam, The Netherlands). The open circuit potential (OCP) values were measured after a stabilization period of 1 h and were recorded in the EIS software before starting the EIS measurements. The EIS scan frequency was ranged from 100 kHz to 0.1 Hz. The EIS experiments were performed under a sinusoidal wave perturbation of ±5 mV and the data were collected using NOVA software (Version 1.8.14, Metrohm Autolab B.V., Amsterdam, The Netherlands) at a rate of 10 points per decade change in frequency.

The morphologies of the coating samples were examined by field emission SEM (model JSM-7400F from JEOL, Tokyo, Japan), and the distributions of the added nanoparticles in the final coatings were observed by EDX. In preparation for SEM, the samples were mounted on the stubs using carbon tape and were coated with platinum by sputtering.

## 3. Results and Discussion

### 3.1. FTIR and XRD Results

**Figure 1:**
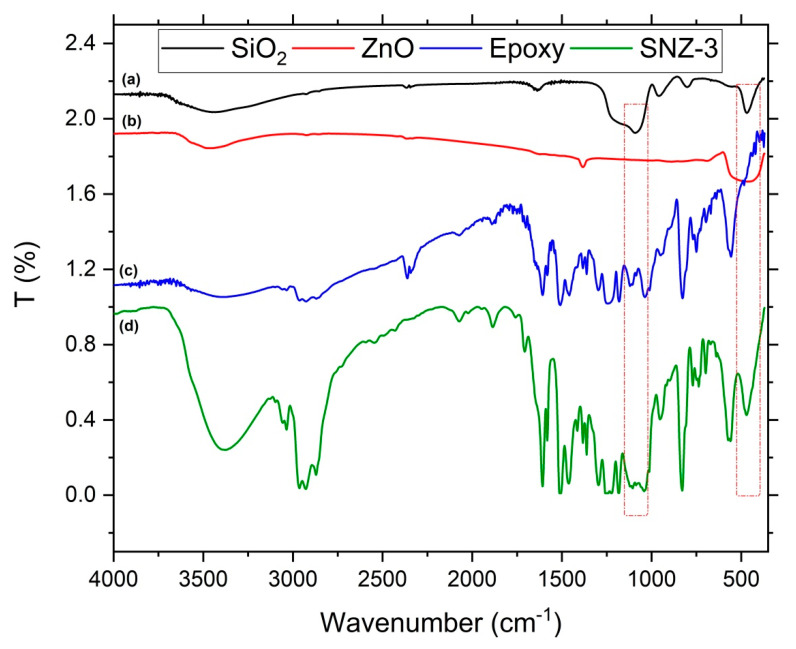
Fourier transform infrared (FTIR) spectra of (**a**) SiO_2_ nanoparticles, (**b**) ZnO nanoparticles, (**c**) epoxy resin, and (**d**) SiO_2_/ZnO-modified epoxy coating (SNZ-3).

Figure 1 shows the FTIR spectra of the SiO_2_ and ZnO nanoparticles, the neat epoxy resin, and the nanoparticle-modified epoxy resin. The spectra were collected over the 400–4000 cm^−1^ range, capturing the changes in the resin spectrum caused by the nanoparticles. The silica nanoparticles presented a characteristic absorption peak at 460 cm^−1^ generated by rocking and stretching vibrations of the Si–O bonds, and another peak at 1100 cm^−1^, which was attributed to internal Si–O–Si stretching vibrations of the SiO asymmetric band [19]. The ZnO nanoparticles yielded a broad spectrum with a clear peak at approximately 460 cm^−1^, which represents Zn–O stretching. The resin matrix yielded peaks in the range of 3340–3200 cm^−1^, possibly arising from NH_2_ vibration absorptions of the amine compounds and OH stretching induced by epoxy cross-linking and ring opening. Other peaks were attributed to the epoxy methane group (3038 cm^−1^), 1,4-substitution of the aromatic ring in the DGEBA resin (830 cm^−1^), and aromatic-ring C–C stretching vibrations (557 cm^−1^). The absorption peak of the terminal epoxy group at 917 cm^−1^ was not observed in our samples, implying that no unreacted epoxy remained in the system. The strong peak at 1247 cm^−1^ represents the ether group (Ar–C–O–C–alkyl) of bisphenol-A in DGEBA epoxy [20]. The presence of silica particles was confirmed in the final SNZ-3 coating, which presented bands at 460 cm^−1^ and 1100 cm^−1^ attributed to Si–O bond stretching and Si–O–Si stretching vibrations, respectively. Both bands were absent in the spectrum of epoxy resin.

**Figure 2:**
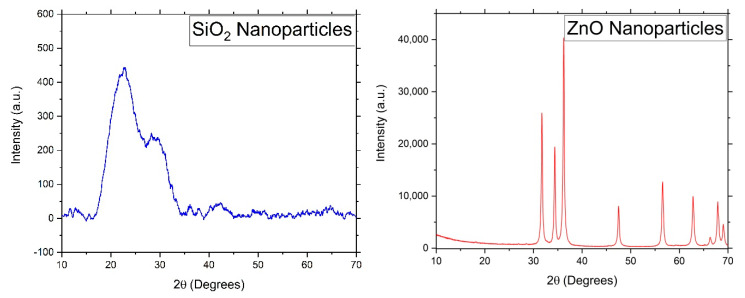
X-ray diffraction (XRD) patterns of pristine silica (**left**) and ZnO (**right**) nanoparticles.

The presence of the ZnO and the SiO_2_ nanoparticles in the prepared coatings was also confirmed by XRD. As ZnO is polycrystalline while SiO_2_ is amorphous, the XRD spectra show the characteristic peaks of the ZnO nanoparticles. The XRD spectra of the pristine SiO_2_ and ZnO nanoparticles are shown in Figure 2. All of the characteristic peaks in these spectra have been reported in the literature [21,22].

**Figure 3:**
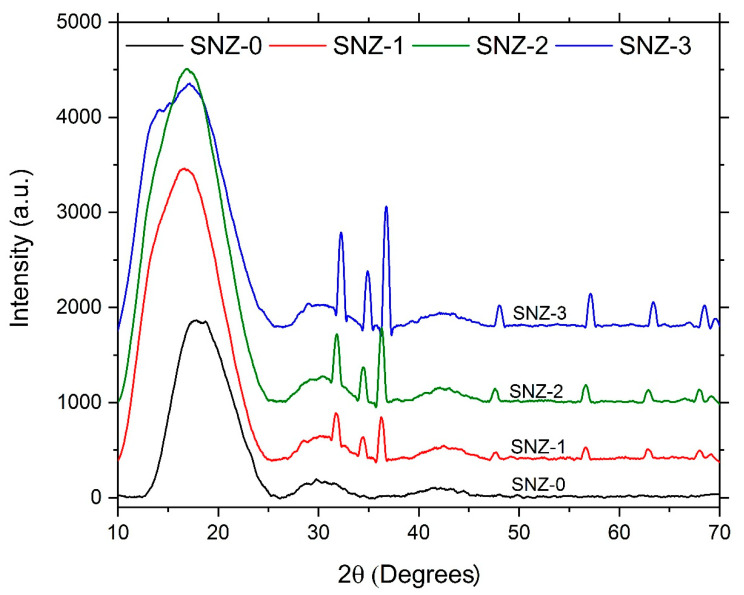
XRD patterns of the SiO_2_/ZnO-incorporated composite epoxy coatings. The composition details of these formulations are provided in Table 1.

Figure 3 shows the X-ray diffractograms of the ZnO-modified epoxy/silica coatings. The multiple peaks in the diffraction patterns of the coatings at 2*θ* = 10–40° can be resolved by deconvolution. Here, the XRD profiles were deconvolved by a Gaussian peak function, and the obtained peaks were analyzed. The *d*-spacing was calculated by Bragg’s law and the crystallite size *d* and the lattice strain values were obtained by Scherrer’s formula. The calculated parameters of the peaks selected from the deconvolution analysis of the XRD profiles are shown in Table 2.

The average d-spacing between the SiO_2_ crystal layers increased when the SiO_2_ nanoparticles were incorporated into the coating matrix. Change in d-spacing helps in determining the dispersion behavior of the filler in the matrix that occurs either by intercalation or exfoliation. Dispersion by exfoliation occurs when the d-spacing is higher than 10 nm [23]. The d-spacing for 5 wt.% nanosilica dispersed in the epoxy matrix was found to significantly increase from 4.52 Å to 5.31 Å, which ensures the dispersion of the nanosilica in the epoxy matrix via intercalation mechanism as reported by Gurusideswar et al. [24]. In contrast, the d-spacing of the ZnO crystal layers was not significantly changed by incorporation into the epoxy coating matrix, or by increasing the ZnO concentration in the epoxy coating matrix. Table 2 shows the varying crystallite sizes of the various crystal structures of SiO_2_ and ZnO when incorporated into the epoxy coating matrix. The overall crystallinity of the prepared epoxy coatings was an increasing function of ZnO concentration. The lattice strains in the SiO_2_ and the ZnO nanoparticles were higher in the epoxy coating matrix than in the bulk nanopowders.

### 3.2. Electrochemical Impedance Spectroscopy

**Figure 4:**
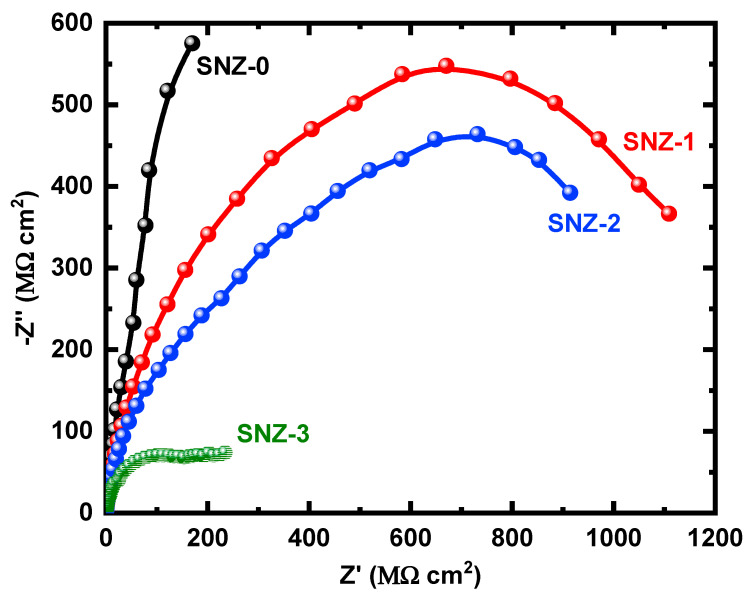
Nyquist plots of the coatings after 1 h immersion in a 3.5% NaCl solution.

**Figure 5:**
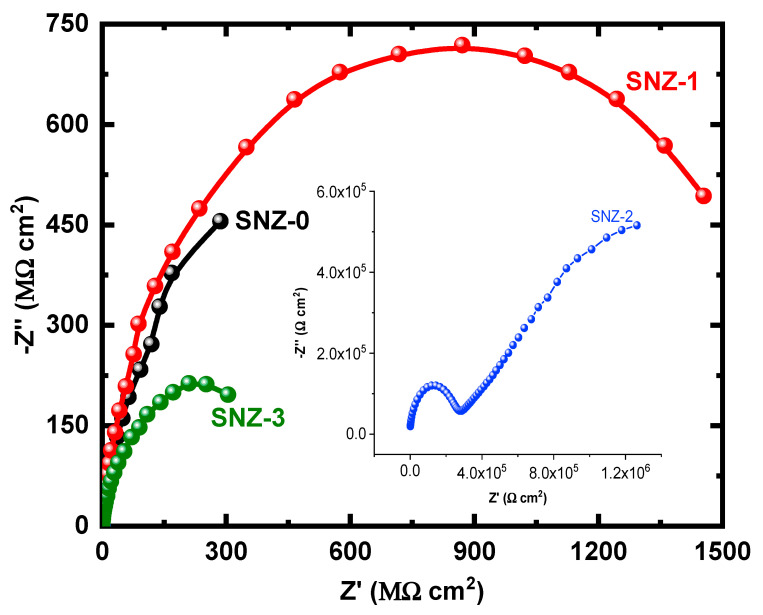
Nyquist plots of the coatings after 5 days’ immersion in a 3.5% NaCl solution.

**Figure 6:**
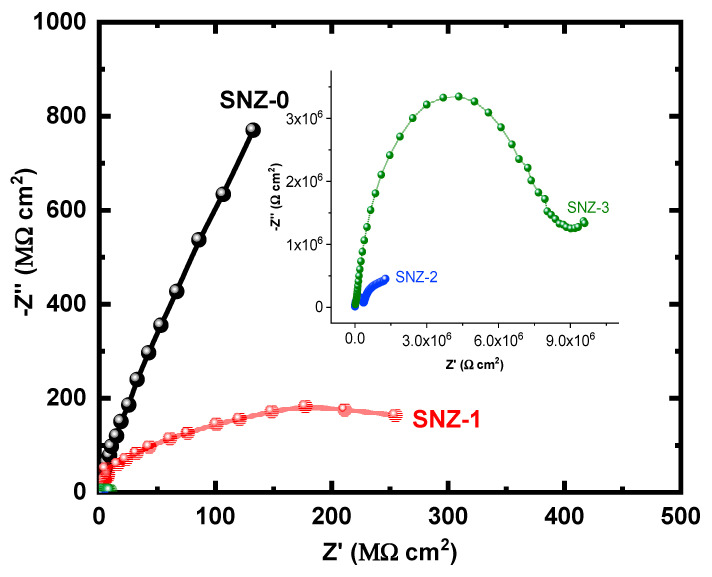
Nyquist plots of the coatings after 10 days’ immersion in a 3.5% NaCl solution.

**Figure 7:**
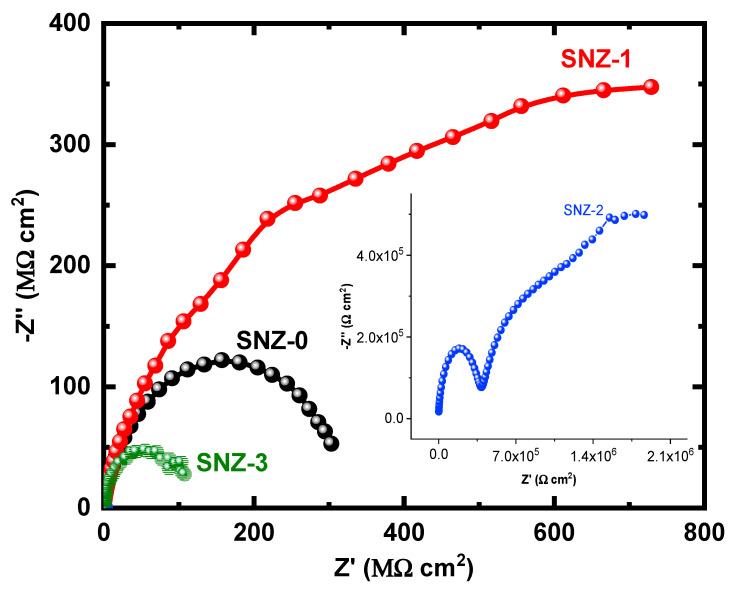
Nyquist plots of the coatings after 15 days’ immersion in a 3.5% NaCl solution.

**Figure 8:**
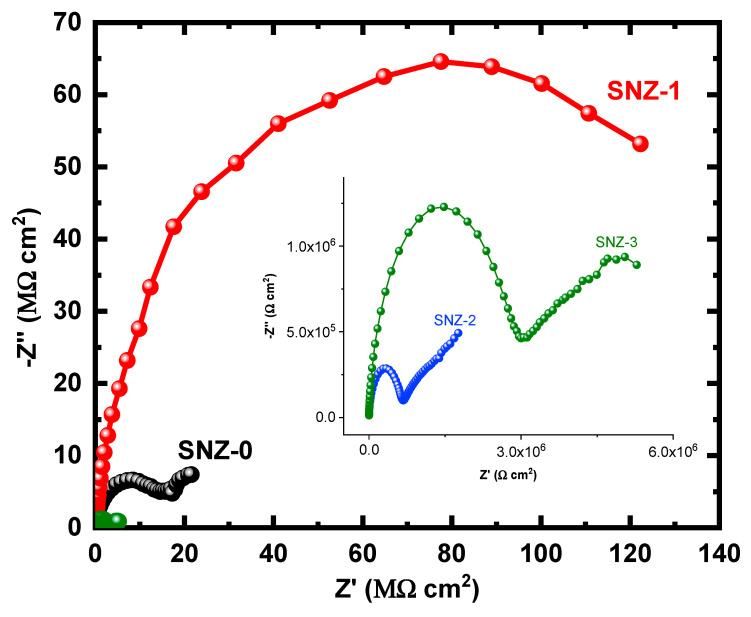
Nyquist plots of the coatings after 20 days’ immersion in a 3.5% NaCl solution.

**Figure 9:**
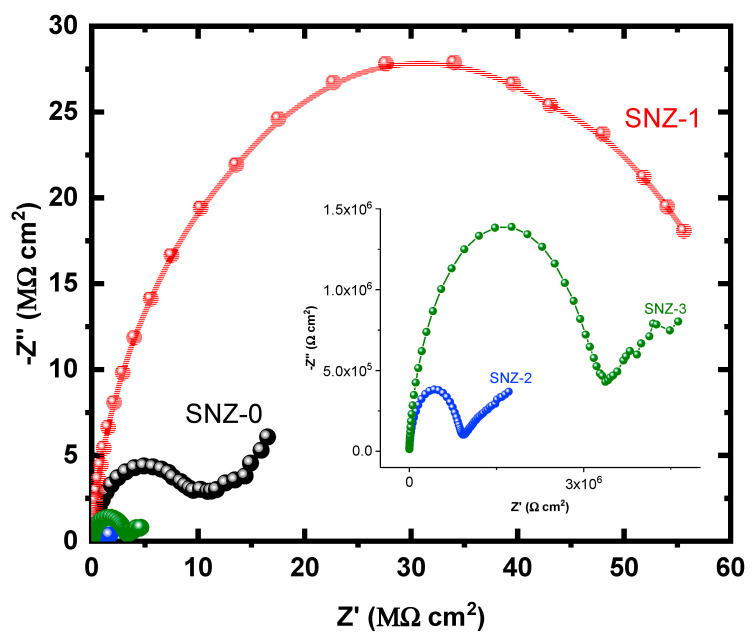
Nyquist plots of the coatings after 25 days’ immersion in a 3.5% NaCl solution.

**Figure 10:**
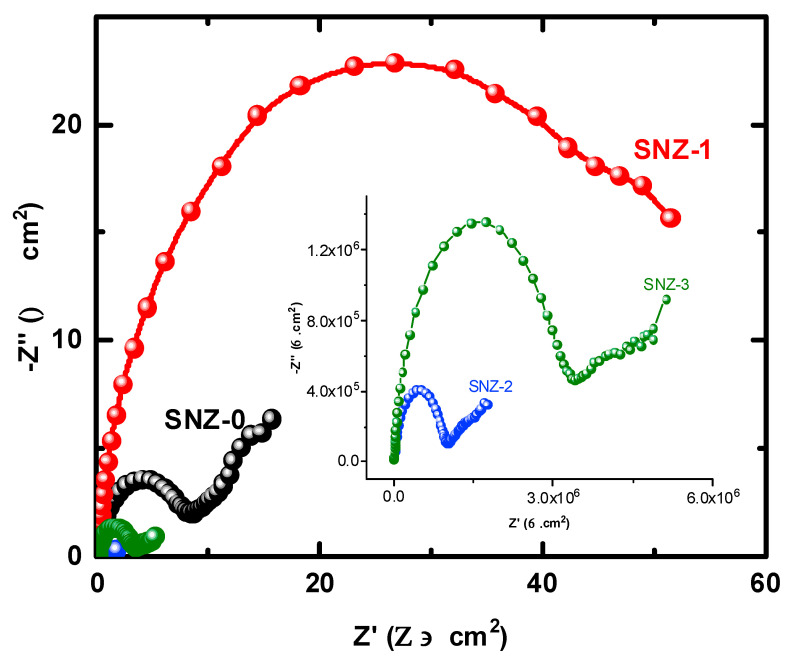
Nyquist plots of the coatings after 30 days’ immersion in a 3.5% NaCl solution.

The EIS measurements evaluate the kinetic parameters associated with the electron transfer reaction at the surface–electrolyte interface and hence reveal the degradation mechanism of the coatings [25,26,27,28,29,30,31]. Figure 4 shows the Nyquist plots of the nanoparticle-incorporated epoxy coatings after immersion for 1 h in a 3.5% saline solution. To better understand the corrosion resistance of the coatings, the effect of 3.5% NaCl exposure on the coatings impregnated with ZnO nanoparticles was examined over extended periods of time. The impedance measurements were carried out after 5, 10, 15, 20, 25, and 30 days, and the corresponding Nyquist plots are shown in Figure 5, Figure 6, Figure 7, Figure 8, Figure 9 and Figure 10. The addition of 1 wt.% ZnO nanoparticles (Coating SNZ-1) to the coating formulation improved the corrosion resistance of the coating optimized for anticorrosion and mechanical properties (with silica nanoparticles), as reported in our earlier studies [17,18]. Adding 1 wt.% ZnO nanoparticles to the formulation containing 5 wt.% SiO_2_ nanoparticles further improved the corrosion resistance of this formulation. The synergistic effect was confirmed by the Nyquist plots of the SNZ-1 coating immersed in a saline solution for various periods. The Nyquist plots also revealed that when added at 2 and 3 wt.%, the ZnO nanoparticles deteriorated the corrosion resistance of the coatings, probably by generating failure sites on the coating surface after long-term exposure to the chloride test solution. The failure sites are the small pinholes in the coatings that appear after curing due to solvent evaporation. In some cases, the removal of agglomerated nanoparticles on the surface also creates failure sites. Such failure sites provide pathways for the diffusion of water molecules into the coating, which degrades the corrosion resistance [32].

**Figure 11:**
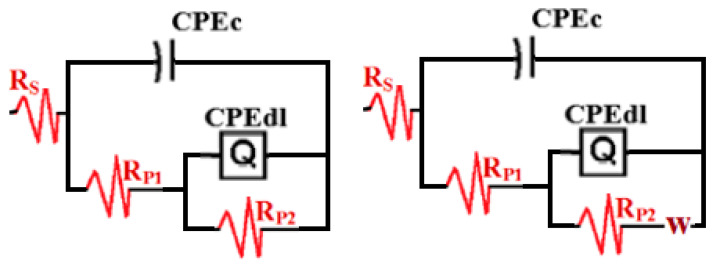
Equivalent electrical circuit models fitted to the obtained impedance.

Figure 11 displays the equivalent electrical circuit models fitted to the impedance data obtained for the SNZ-0, SNZ-1, SNZ-2, and SNZ-3 samples after the various exposure periods of time. The first equivalent circuit of Figure 11 consists of a solution resistance (R_S_), a coating capacitance (CPEc), a polarization resistance (R_P1_), a double-layer capacitance (CPEdl), and a second polarization resistance (R_P2_) [33,34]. The equivalent circuit shown in the second image of Figure 11 is the same but with a Warburg impedance (W) added [33,34]. The values of these elements are listed in Table 3.

It is well known that *R*_P1_ represents the polarization resistance between the interface of the solution and the epoxy coating, and *R*_P2_ is the resistance between the corrosion product layer and the solution [26,27,28,29]. The overall polarization resistance (or coating resistance, expressed in MΩ cm^2^) is obtained by combining *R*_P1_ and *R*_P2_. This value, which represents the overall resistance to ion transport through the coating, is among the most important determining factors of the anticorrosive protection offered by the coating [35]. If the coating has a high R_P2_ (>10^8^ Ω cm^2^) after several days’ exposure to the chloride test solution, it offers excellent corrosion resistance. Any decrease in R_P2_ indicates failure of the coating and consequent formation of a corrosion product below the coating. The protective behavior of coatings is often graded by the following guideline [36,37,38]: excellent (>10^8^ Ω cm^2^), adequate (10^7^–10^8^ Ω cm^2^), doubtful (10^6^–10^7^ Ω cm^2^), or bad (<10^6^ Ω cm^2^). Following this guideline, the SNZ-1 coating formulation exhibited excellent protective behavior and outperformed the other reported coatings.

It is seen from Table 3 that the value of Y_Q_ decreases with the increase in immersion time as well as the presence of ZnO nanoparticles. The value of the “n” component varies for the current samples in the range of 1 > n > 0.5, indicating that the coating layer is very resistive and if a corrosion product is formed, it will have few porosities. Additionally, the closer the value of “n” is to 1, the semicircle is close to ideal capacitance. However, a value of “n” lower than 1 indicates that the semicircle is depressed as well as the presence of a real capacitance included in the circuit. These values of the “n” component indicate that the present constant phase elements (CPEdl, Q) in Figure 11 can be expressed as a double-layer capacitor with some pores. The decrease in the Y_Q_ value is due to the high corrosion resistance against the dissolution of the coatings in the chloride solution. Moreover, the presence of both CPEc and CPE_dl_ gives more information on the passivation of the coating versus corrosion via decreased porosity. However, the presence of W in the equivalent circuit shown in Figure 11 confirms the passivation of the surface through a decrease in the mass transfer.

Prolonging the exposure periods of time is seen to have an effective influence on the behavior of coatings against corrosion in the chloride test solutions. Thus, the Nyquist plots obtained after a short immersion time of 1 h, as seen in Figure 4, show the widest diameters of the semicircles. Increasing the time of immersion to 5 d, as seen in Figure 5, shows smaller diameters and these get even much smaller with further increases in the exposure time periods before measurement. The lowest diameters obtained for all samples are shown in Figure 10, which represents the Nyquist plots obtained for the different coatings after 30 d of immersion in 3.5% NaCl solutions. The decrease in the corrosion resistance over time is most probably recorded due to the degradation of the coatings with time. All impedance data thus confirm that the SZN-1 sample has the highest performance against corrosion even after prolonging the exposure periods of time up to 30 days.

### 3.3. Field-Emission Scanning Electron Microscopy (FE-SEM)

**Figure 12:**
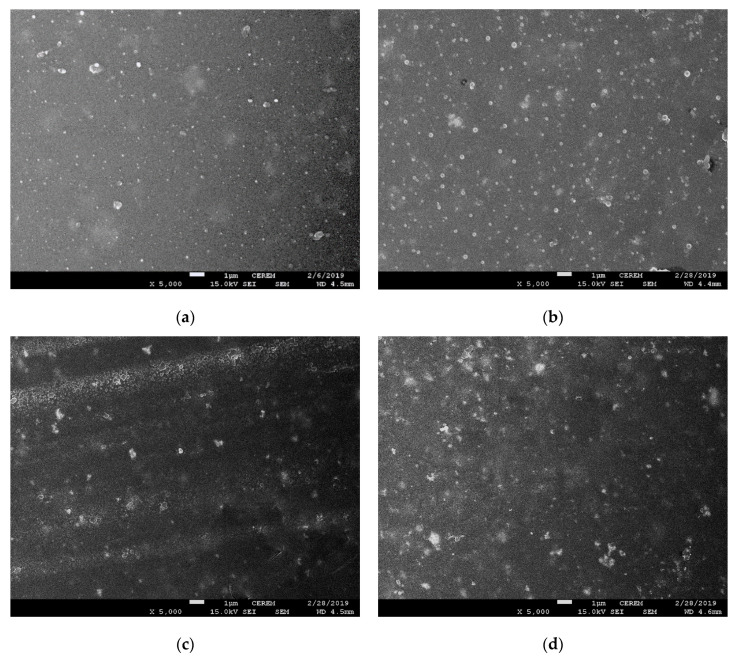
Scanning electron microscopy (SEM) images showing the representative surface morphology of the composite epoxy coatings (**a**) SNZ-0, (**b**) SNZ-1, (**c**) SNZ-2, and (**d**) SNZ-3.

**Figure 13:**
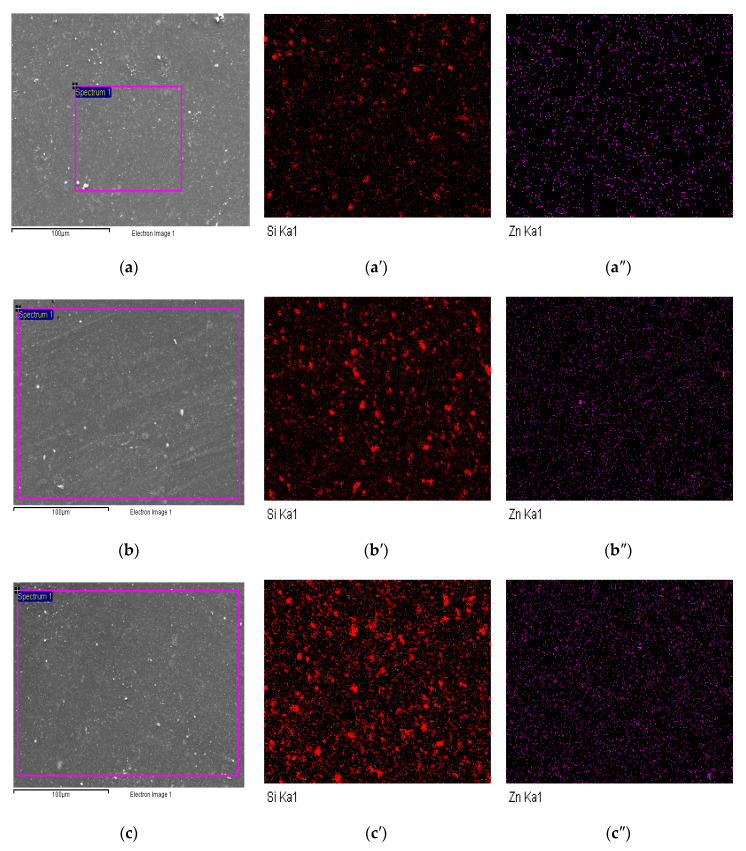
SEM images (**a**–**c**) showing the highlighted area for EDX analysis for elemental mapping, Si elemental mapping results (**a**′–**c**′), and Zn elemental mapping results (**a**″–**c**″) for (**a**) SNZ-1, (**b**) SNZ-2, and (**c**) SNZ-3.

The morphologies and distributions of the nanoparticles in the samples were investigated by field-emission SEM. Figure 12 shows the SEM images of (a) SNZ-0, (b) SNZ-1, (c) SNZ-2, and (d) SNZ-3. The images clearly show the nanoparticles incorporated into the epoxy matrix. The SNZ-1 sample possessed a smooth surface and the nanoparticles were well dispersed throughout the sample. In contrast, the surfaces of the SNZ-2 and SNZ-3 samples were rough and nanoparticle aggregates were visible. The aggregates resulted from high overall loading of the nanoparticles, which became increasingly difficult to disperse. The distributions of the SiO_2_ and ZnO nanoparticles in the epoxy matrix were determined in an EDX analysis of the sample surfaces. The EDX results are presented in Table 4, and Figure 13 shows the area at which EDX was carried out.

The dispersions and distributions of the nanoparticles in the epoxy matrix were confirmed by an elemental mapping analysis. For illustrative purposes, we show the images of the Si and Zn contents. Panels (a), (a′), and (a″) of Figure 13 show the scanned area, the silica nanoparticle contents, and the ZnO nanoparticle contents of the SNZ-1 coating, respectively. Similarly, panels (b), (b′), and (b″) and (c), (c′), and (c″) of Figure 13 present the scan areas, silica nanoparticle contents, and ZnO nanoparticle contents of the SNZ-2 and SNZ-3 coatings, respectively. As shown in the sequence of images Figure 13a′–c′, increasing nano-ZnO increased their aggregation of the nano-SiO_2_ in the coatings. However, the nano-ZnO contents (Figure 13a″–c″) were uniformly distributed in all the coating formulations.

## 4. Conclusions

- Different weight percentages of nano-ZnO along with a fixed percentage of nano-SiO_2_ were incorporated into a DGEBA epoxy resin using the sonication technique. Samples were cured using polyamidoamine adduct hardener (D-450).
- The addition of 1 wt.% nano-ZnO (SNZ-1) in a DGEBA epoxy along with nano-SiO_2_ showed a synergistic effect by achieving a higher corrosion resistance.
- Enhancement in barrier properties and high corrosion resistance performance of the final coating was attributed to the high surface area of the nano-sized ZnO particles.
- The best values for the corrosion resistance of the studied samples were obtained for SNZ-1 samples even after 30 days’ exposure to a 3.5% NaCl solution.
- At higher concentrations of ZnO nanoparticles (2 and 3 wt.%), they agglomerate with the existing nano-SiO_2_, leading to a decrease in the corrosion current density and preventing further improvement of the corrosion resistance of the coatings.

## Figures and Tables

**Table 1:** Compositions (in wt.%) of the nanoparticle-modified formulations.

Formulation Code	Resin	MIBK	Xylene	Modifier	SiO_2_	ZnO	Hardener
SNZ-0	83.34	8	8	2.0	5	0	16.66
SNZ-1	83.34	8	8	2.0	5	1	16.66
SNZ-2	83.34	8	8	2.0	5	2	16.66
SNZ-3	83.34	8	8	2.0	5	3	16.66

**Table 2:** XRD peak parameters of the samples.

Sample	Peak Position (2*θ*)	FWHM	*d*-Spacing (Å)	Crystallite Size (nm)	Lattice Strain
SiO_2_	19.58589	2.94332	4.528828	2.86	0.0744
	22.57793	5.59728	3.934975	1.51	0.1223
ZnO	31.73409	0.23328	2.817422	36.99	0.0036
	34.38974	0.23302	2.605688	37.29	0.0033
	36.22022	0.24532	2.478091	35.6	0.0033
SNZ-0	16.65572	3.81686	5.31838	2.2	0.1138
	19.59351	5.46692	4.527084	1.54	0.1381
SNZ-1	13.38835	3.07641	6.60806	2.72	0.1144
	17.12733	7.5236	5.172983	1.12	0.218
	31.7897	0.54023	2.81262	15.98	0.0083
	34.42681	0.56117	2.602967	15.49	0.0079
	36.28277	0.50856	2.473962	17.18	0.0068
SNZ-2	13.25658	2.67718	6.673446	3.12	0.1005
	17.17584	7.69235	5.158482	1.09	0.2223
	31.8155	0.5878	2.810398	14.68	0.009
	34.46997	0.54238	2.599806	16.02	0.0076
	36.30078	0.55318	2.472776	15.79	0.0074
SNZ-3	13.15965	2.91287	6.722382	2.87	0.1102
	17.32535	8.01852	5.114302	1.05	0.2296
	32.10207	0.33925	2.785961	25.46	0.0051
	34.91708	0.53854	2.567533	16.16	0.0075
	36.75516	0.51518	2.443243	16.98	0.0068

**Table 3:** Electrochemical impedance spectroscopy (EIS) data of the SNZ samples after different immersion times in the chloride test solutions.

Coating	Time	EIS Parameters						
		R_s_ Ω cm^2^	CPEc µF cm^−2^	R_P1_ M Ω cm^2^	CPE_dl_		R_P2_ M Ω cm^2^	W Ω^−^^1/2^
					Y_Q_/µF cm^−2^	n		
SNZ-0	1 h	55.48	2.648 × 10^−9^	5.574	8.999 × 10^−6^	0.64	296.1	-
	5 d	57.96	2.123 × 10^−9^	6.571	2.999 × 10^−6^	0.59	3.493	-
	10 d	58.12	5.815 × 10^−9^	6.987	8.125 × 10^−8^	0.75	4.516	2.222 × 10^−8^
	15 d	58.41	2.333 × 10^−8^	9.298	2.087 × 10^−8^	0.80	5.361	4.358 × 10^−8^
	20 d	58.96	8.087 × 10^−9^	8.112	3.793 × 10^−9^	0.83	14.76	1.787 × 10^−7^
	25 d	59.32	2.421 × 10^−9^	9.501	3.673 × 10^−9^	0.85	9.711	2.800 × 10^−7^
	30 d	59.87	7.64 × 10^−10^	9.685	3.757 × 10^−9^	0.88	7.957	3.810 × 10^−7^
SNZ-1	1 h	42.04	1.233 × 10^−9^	4.496	3.771 × 10^−10^	0.80	109.9	-
	5 d	44.11	5.954 × 10^−9^	8.273	8.831 × 10^−11^	0.74	1760	-
	10 d	48.32	1.321 × 10^−9^	2.281	1.112 × 10^−9^	0.69	36.80	2.222 × 10^−8^
	15 d	49.14	7.49 × 10^−11^	3.243	8.399 × 10^−11^	0.48	1987	4.358 × 10^−8^
	20 d	50.18	1.185 × 10^−9^	6.129	1.746 × 10^−11^	0.80	155.6	1.787 × 10^−7^
	25 d	51.12	1.192 × 10^−9^	5.528	1.923 × 10^−9^	0.76	66.34	2.800 × 10^−7^
	30 d	52.87	1.287 × 10^−9^	5..691	1.296 × 10^−8^	0.71	6.961	3.810 × 10^−7^
SNZ-2	1 h	54.04	8.350 × 10^−9^	8.701	1.471 × 10^−9^	0.67	858.0	-
	5 d	56.23	9.07 × 10^−10^	8.962	6.845 × 10^−8^	0.63	448.3	-
	10 d	55.81	9.52 × 10^−10^	9.180	4.762 × 10^−7^	0.63	1.277	4.611 × 10^−6^
	15 d	56.24	9.60 × 10^−10^	9.319	7.006 × 10^−7^	0.54	1.506	6.735 × 10^−6^
	20 d	54.98	9.70 × 10^−10^	9.398	1.452 × 10^−6^	0.26	1.355	1.826 × 10^−7^
	25 d	56.39	9.91 × 10^−10^	9.562	1.578 × 10^−6^	0.21	2.131	4.316 × 10^−7^
	30 d	57.54	9.91 × 10^−10^	9.846	1.365 × 10^−6^	0.17	2.659	4.612 × 10^−7^
SNZ-3	1 h	57.81	1.126 × 10^−9^	8.927	2.713 × 10^−9^	0.69	181.4	-
	5 d	57.96	1.321 × 10^−9^	9.011	7.01 × 10^−10^	0.67	2.369	-
	10 d	58.01	7.26 × 10^−10^	9.269	9.140 × 10^−8^	0.59	2.911	1.311 × 10^−8^
	15 d	58.99	3.652 × 10^−9^	9.289	1.589 × 10^−7^	0.51	2.847	1.682 × 10^−8^
	20 d	59.18	1.240 × 10^−9^	9.347	3.008 × 10^−7^	0.47	2.449	5.012 × 10^−9^
	25 d	58.78	1.19 × 10^−10^	9.521	1.238 × 10^−8^	0.48	3.794	1.841 × 10^−8^
	30 d	59.41	1.208 × 10^−9^	9.693	1.626 × 10^−8^	0.44	3.898	2.314 × 10^−6^

**Table 4:** Elemental composites of the prepared coatings and their weight percentages, detected by energy-dispersive X-ray spectroscopy (EDX).

Sample Code	Nanoparticles	Elements	Percentages (wt.%)
SNZ-0	SiO_2_	C, O, Si	76.24, 21.41, 2.34
SNZ-1	SiO_2_, ZnO	C, O, Si, Zn	75.03, 21.62, 2.27, 1.08
SNZ-2	SiO_2_, ZnO	C, O, Si, Zn	75.50, 20.40, 2.27, 1.83
SNZ-3	SiO_2_, ZnO	C, O, Si, Zn	74.74, 20.24, 2.23, 2.79
